# miR-294 and miR-410 Negatively Regulate *Tnfa*, Arginine Transporter *Cat*1/2, and *Nos*2 mRNAs in Murine Macrophages Infected with *Leishmania amazonensis*

**DOI:** 10.3390/ncrna8010017

**Published:** 2022-02-06

**Authors:** Stephanie Maia Acuña, Jonathan Miguel Zanatta, Camilla de Almeida Bento, Lucile Maria Floeter-Winter, Sandra Marcia Muxel

**Affiliations:** 1 Departamento de Fisiologia, Instituto de Biociências, Universidade de São Paulo, São Paulo 05508-090, SP, Brazil; jonathanzanatta@usp.br (J.M.Z.); camillabento@usp.br (C.d.A.B.); lucile@usp.br (L.M.F.-W.); 2 Departamento de Imunologia, Instituto de Ciências Biomédicas, Universidade de São Paulo, São Paulo 05508-020, SP, Brazil

**Keywords:** microRNA, macrophage, *Leishmania*, nitric oxide synthase 2, *Cat*2*/Slc*7*a*2, *Tnfa*

## Abstract

MicroRNAs are small non-coding RNAs that regulate cellular processes by the post-transcriptional regulation of gene expression, including immune responses. The shift in the miRNA profiling of murine macrophages infected with *Leishmania amazonensis* can change inflammatory response and metabolism. L-arginine availability and its conversion into nitric oxide by nitric oxide synthase 2 (*Nos*2) or ornithine (a polyamine precursor) by arginase 1/2 regulate macrophage microbicidal activity. This work aimed to evaluate the function of miR-294, miR-301b, and miR-410 during early C57BL/6 bone marrow-derived macrophage infection with *L. amazonensis*. We observed an upregulation of miR-294 and miR-410 at 4 h of infection, but the levels of miR-301b were not modified. This profile was not observed in LPS-stimulated macrophages. We also observed decreased levels of those miRNAs target genes during infection, such as Cationic amino acid transporters 1 (*Cat*1/*Slc*7*a*1), *Cat*2*/Slc*7*a*22 and *Nos*2; genes were upregulated in LPS stimuli. The functional inhibition of miR-294 led to the upregulation of *Cat*2 and *Tnfa* and the dysregulation of *Nos*2, while miR-410 increased *Cat*1 levels. miR-294 inhibition reduced the number of amastigotes per infected macrophage, showing a reduction in the parasite growth inside the macrophage. These data identified miR-294 and miR-410 biomarkers for a potential regulator in the inflammatory profiles of microphages mediated by *L. amazonensis* infection. This research provides novel insights into immune dysfunction contributing to infection outcomes and suggests the use of the antagomiRs/inhibitors of miR-294 and miR-410 as new therapeutic strategies to modulate inflammation and to decrease parasitism.

## 1. Introduction

MicroRNAs (miRNAs) are small non-coding RNAs with 18–25 nucleotides that mediate the post-transcriptional regulation of the genes involved in physiological and pathological conditions, including the inflammatory response [1,2]. miRNAs are transcribed from intergenic, exonic, or intronic regions by the RNA polymerase II and are processed into primary miRNA transcripts (pri-miRNA), and they subsequently enter the precursor (pre-miRNA) and mature form across a series of steps involving class 2 RNAse III DROSHA and Dicer [3,4]. miRNAs recognize the complementary sequences in the 3′ untranslated regions (3′UTR) of given transcripts, which cause their degradation or translational repression [3,4]. miRNA dysregulation occurs through various mechanisms, encompassing perturbations of miRNA transcription, epigenetic mechanisms, and disruption of the miRNA synthesis machinery [5].

Different miRNA expressions display macrophage polarization during infection, affecting the macrophage activation and the polarization to classically characterized M1-pro-inflammatory or M2-anti-inflammatory/pro-resolution phenotype [6]. The regulation of cytokine/chemokine production, such as by interferon-gamma (IFN-γ), tumor necrosis factor-alpha (TNF-α), and granulocyte-macrophage colony-stimulating factor (GM-CSF) as well as the activation of Toll-like receptor (TLR), leads to macrophage differentiation into the M1 phenotype, which increases nitric oxide synthase 2 (NOS2) expression and NO production with microbicidal activity. Moreover, interleukin 4 (IL-4), IL-13, transforming growth factor-beta (TGF-β), IL-10, and macrophage colony-stimulating factor (M-CSF) differentiate macrophages into the M2 phenotype, expressing higher levels of arginase 1 (ARG1) and polyamine production [7].

Leishmaniasis is a neglected tropical disease that is caused by the *Leishmania* parasite, which manifests through a broad range of clinical outcomes, from cutaneous to visceral disease, depending on the species that is infecting the host. *Leishmania amazonensis* causes a cutaneous disease [8,9,10]. The parasite switches between an invertebrate host (sand fly *Phlebotomus* or *Lutzomyia)* and vertebrate hosts, such as mammals, rodents, and humans. The infected sand fly regurgitates the promastigotes form of *Leishmani*a during the bite, which is recognized and phagocytosed by the resident macrophages, and it differentiates into the amastigotes form [11,12,13]. *Leishmania* is dependent on the host’s L-arginine metabolism for polyamines to proliferate and establish a successful infection [14,15,16]. However, the host’s immune response also uses this amino acid to produce NO, leading to parasite clearance [17,18]. In this case, the amino acid used can indicate how the macrophages polarize on the M1/M2 dichotomy [14,19]. In this scenario, miRNAs can act as regulators of how the cells decide to use the amino acid.

Over the past two decades, the regulation of the physiological and pathophysiological processes mediated by miRNAs has been widely studied, including in cancer studies and in studies on inflammatory and infectious diseases [5,20,21,22,23], as has the identification of miRNAs as a biomarker for infection and for the prognosis of diseases [24,25,26]. Our previous data revealed that the entrance of the parasite into the macrophages initiated the expression of miRNAs. miR-294 was upregulated after 4 and 24 h of infection with BMDM from BALB/c mice [27]. Additionally, the expression of that miRNA was inversely proportional to the expression of *Nos*2/NOS2, suggesting that they could be interacting with the *Nos*2 3′UTR. We demonstrated that this miRNA could bind to the 3′UTR portion of the mRNA. Functional inhibition led to an overexpression of *Nos*2*/*NOS2 [27], which plays a key role in the macrophage polarization and leads to susceptibility to the infection. Later, we demonstrated that the treatment of these macrophages with melatonin regulated not only the miR-294 expression but also the *Nos*2 and *Tnfa* transcripts [3]. After that, we showed that miR-294 expression was independent of TLR-2, TLR-4, or MyD88 in C57BL/6J-macrophages infected with *L. amazonensis* [28]. Those data suggest a role of miR-294 in the regulation of pro-inflammatory response of macrophages, guiding us to evaluate miR-294 function in C57BL/6J murine macrophages, a resistant model for *L. amazonensis* infection both in vitro and in vivo [29,30,31,32,33].

This study analyzes how miR-294 and two other miRNAs, miR-301b and miR-410, can target *Nos*2, *Arg*1, *Odc*1, *Slc*7*a*1 *Slc*7*a*2, and *Tnfa* mRNAs and regulate L-arginine metabolism-related genes during C57BL/6 macrophage infection with *L. amazonensis*. Our findings revealed that *L. amazonensis* upregulated miR-294 and miR-410 but not miR-301b after 4 h of infection. On the other hand, the *Slc*7*a*1 L-arginine transporter was downregulated during the infection, and *Nos*2, *Arg*1, *Odc*1, *and Slc*7*a*2 were expressed at similar levels in uninfected macrophages. LPS stimulation presented a distinct profile with reduced miRNAs and increased *Nos*2, *Arg*1 *Slc*7*a*1, *Odc*1, *Arg*22 *Slc*7*a*2, and *Tnfa*. In addition, the functional inhibition of miR-294 upregulated the levels of *Slc*7*a*2 and *Tnfa* and dysregulated *Nos*2 levels, reducing the parasite growth inside the macrophages. Additionally, the functional inhibition of miR-410 upregulated *Slc*7*a*1 mRNA levels, showing a new miRNA–mRNA interaction that can impact infectivity. Our data bring perspectives on the function of the altered expression of miR-294 and miR-410 upon *L. amazonensis* infection and on the existence of miRNAs as biomarkers, which shift the inflammation and impact parasite growth.

## 2. Results

### 2.1. miR-294-3p, miR-301b-3p, and miR-410-3p Target the L-Arginine Metabolic Pathway

**Figure 1:**
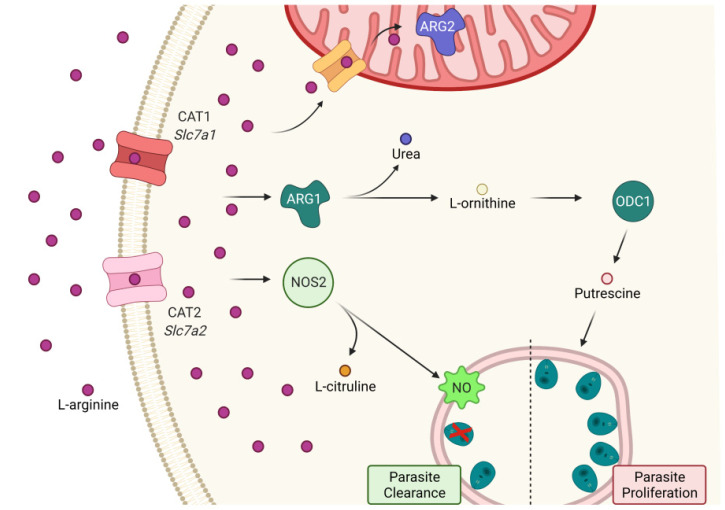
MicroRNAs can regulate the inflammatory branch of L-arginine uptake and consumption of infected macrophages with *Leishmania amazonensis*. Cells use L-arginine as one of the markers of macrophage polarization into the M1 or M2 phenotypes. During infection with *L. amazonensis*, macrophages obtain L-arginine via Cationic Amino Acid Transporters 1 and 2 (CAT1 and CAT2, encoded by the Solute Carrier Transporter family members *Slc*7*a*1 and *Slc*7*a*2). Once inside, the amino acid can be used by Nitric Oxide Synthase 2 (NOS2) to produce the NO free radical, which kills the parasite when at high levels. L-citrulline is a side product of this reaction. Another use of L-arginine is its metabolism into urea and L-ornithine via Arginase 1 (ARG1). Once converted into L-ornithine, it starts the polyamine synthesis pathway, of which Ornithine Decarboxylase 1 (ODC1) is the first branch enzyme. It is accepted that polyamines promote *Leishmania* survival and proliferation. L-arginine can also be transported to the mitochondrion, where it is also converted to urea and L-ornithine. However, the effects of this mitochondrial L-ornithine production are still poorly understood in the inflammatory context. This panel was created with BioRender.com online toll.

It is well-known that L-arginine metabolism contributes to macrophage polarization to the M1 or M2 phenotype. We have already demonstrated that macrophage infection with *L. amazonensis* promotes alterations to this scenario, leading to an M2-like macrophage phenotype in the early stages of infection [34]. To understand the impact of miRNAs on L-arginine metabolism in C57BL/6 macrophages, we focused on the analysis of transcript levels of two arginine transporters (*Slc*7*a*1 and *Slc*7*a*2—Cationic Amino Acids 1 and 2, respectively), Arginases 1 and 2 (*Arg*1 and *Arg*2), Nitric Oxide Synthase 2 (*Nos*2), and Ornithine Decarboxylase 1 (*Odc*1). Together, these genes are involved in the uptake and the first step of arginine metabolization to produce NO or polyamine biosynthesis (Figure 1).

**Figure 2:**
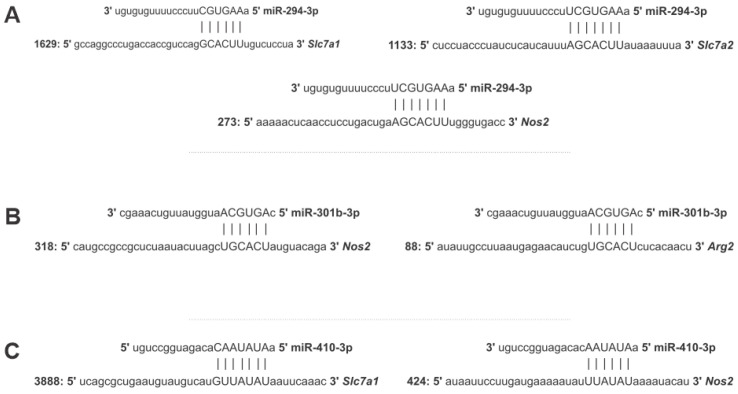
In silico microRNA binding prediction in the 3′UTR region of genes involved in L-arginine metabolism. The search for the prediction of miRNA target repression of genes *Nos*2, *Slc*7*a*1, *Slc*7*a*2, and *Arg*2 using miRmap (https://mirmap.ezlab.org/ accessed on 28 August 2020) [35]; selecting the *Mus musculus* species and mRNAs and setting the combination of ΔG open > 9 (mRNA opening free energy), exact probability > 60 (exact distribution of site over-representation probability), and miRmap score > 60; and identified the binding of (**A**) miR-294-3p, (**B**) miR-301b-3p, and (**C**) miR-410-3p.

Using in silico analysis, we found miRNAs, such as miR-294-3p, miR-301b-3p, and miR-410-3p, binding onto the 3′UTR of those gene transcripts, indicating an intricate network of post-transcriptional gene regulation in L-arginine uptake by transporters and their metabolization beyond just NO production, as we observed previously for miR-294-3p [27]. Focusing on the inflammatory branch of this pathway, in Figure 2A–C, we showed the predicted binding sites of miR-294-3p, miR-301b-3p, and miR-410-3p.

Despite the binding capacity at the *Nos*2 3′UTR, miR-294-3p can also bind to both the *Slc*7*a*1 and *Slc*7*a*2 transporter 3′UTRs, as shown in Figure 2A. On the other hand, miR-301b can bind to the *Nos*2 and *Arg*2 3′UTR (Figure 2B). miR-410-3p can bind to the 3′UTR of *Slc*7*a*1 and *Nos*2 (Figure 2C). Based on experimentally validated and predictive evidence found through in silico analysis, we hypothesized that miR-294, miR-301b, and miR-410 could modulate gene expression and infection control in the early phase of in vitro infection. Furthermore, miR-294 can be a pivotal element regulating this balance by binding in the *Nos*2 mRNA-3′UTR and regulating NOS2 protein expression and NO production. Predictive analysis of mRNA-3′UTR of *Nos-*2, *Arg*2, *Slc*7*a*1, and *Slc*7*a*2 showed a putative binding of miR-301b and that miR-410 can bind to *Nos*2.

### 2.2. Expression of L-Arginine Metabolic Pathway and miR-294-3p, miR-301b-3p, and miR-410-3p

**Figure 3:**
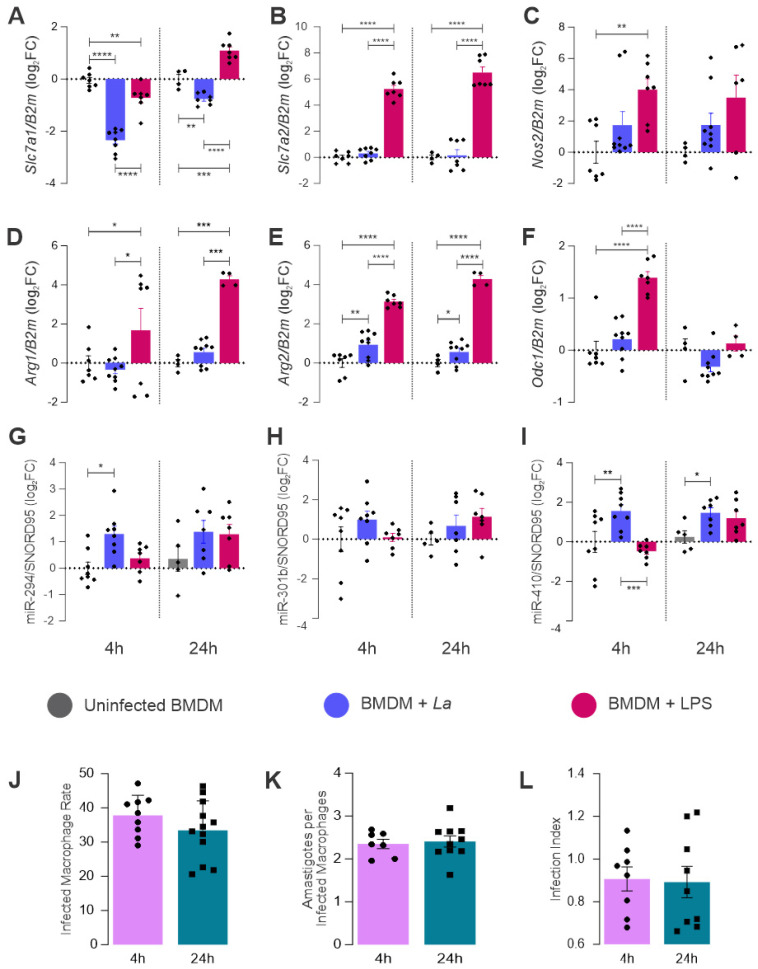
Quantification of L-arginine metabolism-related gene transcripts and miRNA levels on macrophages infected with *L. amazonensis*. Bone Marrow Derived-Macrophages were infected with stationary phase *L. amazonensis* promastigotes (MOI 1:5) or treated with LPS for 4 and 24 h. The total RNA was extracted and converted into cDNA to quantify transcripts of genes related to L-arginine metabolism through RT-qPCR: (**A**) *Slc*7*a*12 (**B**) *Slc*7*a*22 (**C**) *Nos*2, (**D**) *Arg*12 (**E**) *Arg*22 *and* (**F**) *Odc*1. The miRNAs levels were assessed through RT-qPCR: (**G**) miR-294, (**H**) miR-301b (**I**), and miR-410. The infected BMDM were fixed and Giemsa stained to assess the number of total macrophages (*n* = 600), infected macrophages and amastigotes. (**J**) infected macrophage rate [(infected macrophage/total macrophage) ∗ 100], (**K**) amastigotes per infected macrophage, and the product of infected macrophages rate with amastigotes per infected macrophage; (**L**) infection index (rate of infected macrophages multiplied by number of amastigotes per infected macrophage). Statistics: Each dot represents a biological replicate from three independent experiments. Mixed-effects analysis, post hoc test: Sidak’s multiple comparisons. *p*-values: *, *p ≤* 0.05. **, *p* ≤ 0.001, ***, *p* ≤ 0.0005. ****, *p* ≤ 0.0001.

To understand whether the genes involved in L-arginine metabolism and miR-294, miR-301b, and miR-410 are expressed during the infection, we quantified the mRNA levels of genes involved in L-arginine metabolism and miRNAs in BMDM from B6 mice infected with *L. amazonensis* and compared it to LPS, a well-known M1 inductor (Figure 3).

The *L. amazonensis* macrophage infection decreased the levels of *Slc*7*a*1 mRNA after 4 h of infection, and the decreased values were sustained at 24 h, although they were slightly higher than they were at 4 h compared to uninfected macrophages (Figure 3A). In addition, the LPS stimulation also decreased the *Slc*7*a*1 mRNA levels at 4 h, but it was augmented at 24 h compared to uninfected and infected macrophages. We analyzed whether the *Slc*7*a*2 transporter was regulated, but we observed that it was not modulated during infection, remaining the same as it was in the uninfected control. However, LPS stimulation revealed that these macrophages respond to inflammatory stimuli, leading to an increase in the *Slc*7*a*2 transcript levels after 4 and 24 h of LPS stimulation (Figure 3B). The LPS promoted a similar increase of *Nos*2 at 4 h, but it was not modified during infection (Figure 3C).

It was expected that *Arg*1 would decrease to support *Nos*2 L-arginine consumption when receiving an inflammatory stimulus. We observed that LPS stimulation increased *Arg*1 expression, which was not observed during infection (Figure 3D). However, the infection did not increase or decrease, showing similar behavior to the arginine transporter (*Slc*7*a*2). The mitochondrial isoform of Arginase (*Arg*2) is also slightly regulated by infection with *L. amazonensis* at 4–24 h and LPS stimulation at 4 and 24 h. Although the inflammatory stimulus is a signal to increase *Arg*2 transcription, the parasite signalization was not as high as the LPS in both of the tested periods (Figure 3E). Ornithine decarboxylase is the first enzyme committed to polyamine production from L-arginine metabolism. It was expected that if the inflammatory branch of the L-arginine metabolism pathway were not working, then the anti-inflammatory one would be, increasing the polyamine production. However, we did not observe an increase in the *Odc*1 transcription during infection (Figure 3F). Even in the LPS stimulation, it was increased after 4 h.

Regarding miRNA expression, we observed that the infection promoted an increase in the level of miR-294 after 4 h of infection, sustaining the same expression level at 24 h. However, LPS stimulation did not promote a modulation at 4–24 h (Figure 3G). The infection and LPS stimulation did not modulate the miR-301b levels (Figure 3H) significantly.

The infected macrophages showed an increase in the levels of miR-410, while the LPS-stimulated ones showed a significant reduction at 4 h compared to infected individuals (Figure 3I). After 24 h, the infection led to sustained miR-410 expression. The infection led to a faster response when increasing miR-294 and miR-410; this effect was not observed during the LPS stimulation, as there was a classical M1 activator that did not interfere with these miRNAs in the studied periods.

The microscopical quantification of infection showed similar levels of infected macrophages, numbers of amastigotes per infected macrophage, and the infection index (Figure 3C), as previously shown by Muxel [28].

### 2.3. miRNAs Targeting mRNA from Arginine Metabolism Shift the Macrophage Response to Leishmania

**Figure 4:**
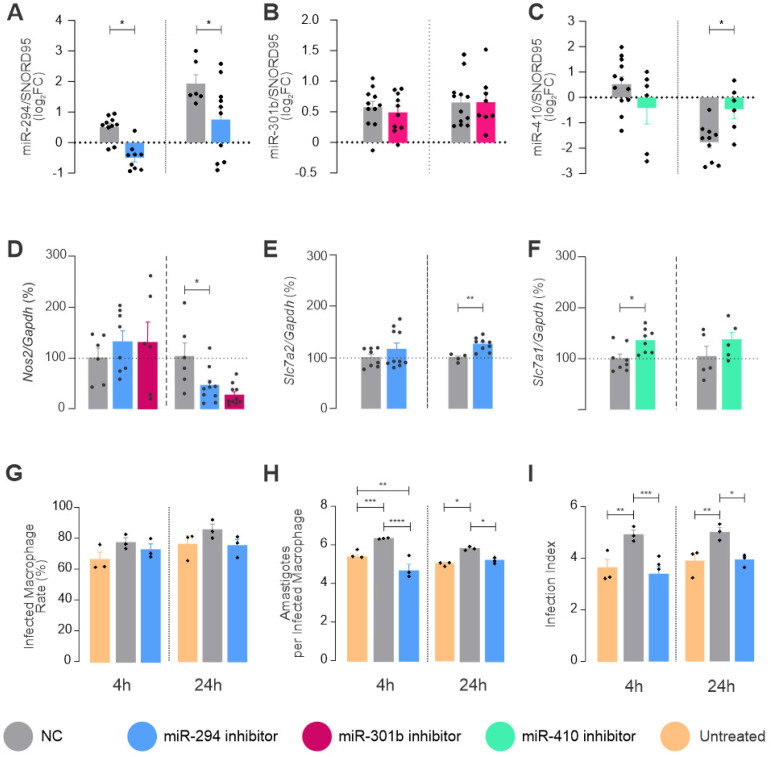
miRNA functional inhibition. Bone marrow derived macrophages were transiently transfected with specific miRNA inhibitors or negative control (NC) and were then infected with *L. amazonensis* for 4 or 24 h. Total RNA was extracted to quantify miRNA levels: (**A**) miR-294, (**B**) miR-301b, and (**C**) miR-410; Or to assess the levels of mRNA targets after inhibition: (**D**) *Nos*2, (**E**) *Slc*7*a*22 and (**F**) *Slc*7*a*1. The infected BMDM were fixed and Giemsa stained to assess the number of total macrophages (*n* = 300), infected macrophages and amastigotes: (G) infected macrophage rate [(infected macrophage/total macrophage) ∗ 100], (**H**) amastigotes per infected macrophage, (**I**) infection index. Statistics: Each dot represents a biological replicate from three independent experiments. Mixed-effects analysis, post hoc test: Sidak’s multiple comparisons. *p*-values: *, *p ≤* 0.05. **, *p* ≤ 0.001, ***, *p* ≤ 0.0005. ****, *p* ≤ 0.0001.

Based on miRNA binding site prediction and miRNA modulation during C57BL/6 macrophage infection with *L. amazonensis*, we performed a miRNA inhibition assay for the miR-294, miR-301b, and miR-410 sequences in order to understand their functions in the infection context (Figure 4). The transfection with negative control was used as the baseline for evaluating the effect of the control and experimental miRNA inhibitor on the expression of miRNA and target-genes for all of the tested times. The miR-294 inhibitor led to a consistent decrease in the miRNA expression at 4 h of infection, which was sustained at 24 h. However, miR-301b did not respond to the inhibition at any tested time (Figure 4A). miR-410 tended to reduce its expression after 4 h of infection, but the inhibition did not significantly decrease the miRNA amount. However, miR-410 had a higher expression than the negative control after 24 h of infection (Figure 4A).

Since we chose miRNAs with the predicted binding site of the 3′UTR of the target genes *Nos*2, *Cat*2/*Slc*7*a*2, and *Cat1*/*Slc*7*a1* and because the genes were increased during LPS stimulation but not during infection, it was expected that inhibition would increase the expression of the target mRNAs. We observed this phenomenon in two of the tested target genes. The functional inhibition of miR-294 and miR-301b increased the expression of *Nos*2 levels slightly at 4 h of infection, which was not significant. Despite that, the inhibition of miR-294 and miR-301b significantly reduced *Nos*2 levels at 24 h compared to the negative control (Figure 4B), showing the role of these miRNAs in the dysregulation of *Nos*2 mRNA. miR-294 also targets *Slc*7*a*2. We monitored whether the infection would promote an increase in the number of transcripts of the transporter in miR-294 inhibition and found a significant increase after 24 h of infection.

On the other hand, the arginine transporter variant *Slc*7*a1* is a target of miR-410, and miR410-inhibition increased the expression of *Slc*7*a1* after 4 h of infection compared to the negative control (Figure 4B).

Regarding the infectivity parameters (Figure 4C), we studied the inhibition of miR-294 compared to the negative control, but the negative control itself promoted a slight increase in the infected macrophage rate and the number of amastigotes per infected macrophage. The infection index was statistically significant when compared to untreated macrophages. On the other hand, treatment with the miR-294 inhibitor promoted a reduction in the number of amastigotes per infected macrophage, reducing the infectivity index at 4 and 24 h compared to the negative control.

### 2.4. miR-294 and miR-301b Can Also Interact with Other Inflammatory Factors during Macrophage Infection with L. amazonensis

**Figure 5:**
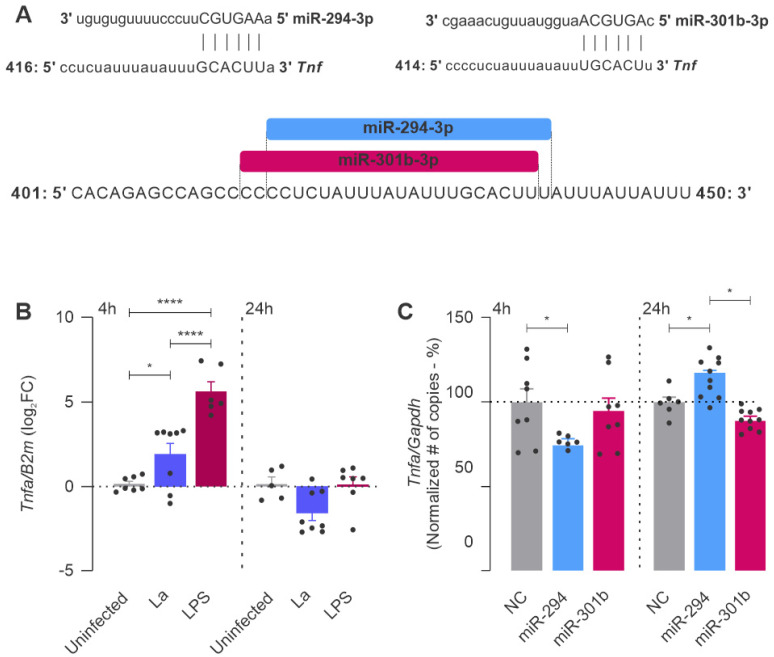
*Tnfa* is an miR-294 and miR-301b target. (**A**) In silico binding site prediction of miR-294 and miR-301b in the *Tnfa* 3′UTR. (**B**) Basal levels of *Tnfa* after *L. amazonensis* infection or LPS stimulation. (**C**) Quantification of the infection’s ability to produce *Tnfa* after miRNA inhibition. Statistics: Each dot represents a biological replicate from three independent experiments. Mixed-effects analysis, post hoc test: Sidak’s multiple comparisons. *p*-values: *, *p ≤* 0.05; ****, *p* ≤ 0.0001.

Inflammatory stimuli can trigger some signaling cascades, the function of which is to regulate the response, both positively and negatively. M1 macrophages produce TNF (tumor necrosis factor), an innate cytokine that promotes inflammatory responses. The prediction analysis showed a putative binding of miR-294 and miR-301 into the 3′UTR of *Tnfa* (Figure 5A).

*Leishmania* infection promoted *Tnfa* expression compared to uninfected macrophages, but at lower levels than LPS stimulation at 4 h (Figure 5B). However, after 24 h, the pathway seemed to stop since the LPS group remained at the same level as the uninfected control (Figure 5B). We observed that the functional inhibition of miR-294 decreased the level of *Tnfa* at 4 h and increased it at 24 h of infection compared to the negative control (Figure 5C). However, miR-301b inhibition only decreased the *Tnfa* levels at 24 h compared to miR-294-inhibition.

## 3. Discussion

Macrophages need to control their recognition processes and signaling cascade to promote an adequate immune response against pathogens [36]. miRNAs can act as powerful tools for this. Some microorganisms can subvert the profile state or the induction of the microbicidal activity of immune cells, as observed in cancers, inflammatory disorders, and infectious diseases caused by viruses, bacteria, fungi, and parasites [5,20,21,23].

The involvement of *Leishmania* factors impacting miRNAs and subverting macrophage responses, such as glycoprotein GP63 of *Leishmania donovani* targeting the host-Dicer1, results in the downregulation of miR-122 and an increase in the parasite burden in the mouse liver [37], which can globally affect the host-miRNAs and post-transcriptional regulation of gene expression. Indeed, *L. amazonensis*-arginase indirectly interferes with an increase of miR-294 and miR-721, targeting *Nos*2 and NO production in BALB/c murine macrophages [27]. Infection with *L. amazonensis* induces miR-30e and miR-302d, which regulate *Nos*2 and NO levels and miR-294 and miR-302d, which regulate *Tnfa* levels, in BALB/c-BMDM [38]. *L. major*-infected human macrophages present with the dysregulation of miRNAs and their corresponding chemokine targets [39]. In *L. major* infection of human macrophages, hypoxia-inducible factor-1 (HIF-1α) regulates miR-210 levels, linking HIF-1α to susceptibility in other species, such as *L. donovani* and *L. amazonensis* [40,41]. miR-146a overexpression downregulates M1 markers such as *Tnfa* and *Nos*2, increasing *Arg*1 and IL-10 in *L. donovani*-infected BMDM [42]. Let-7e interferes in TLR, NFkB, IRF, and MAPK signaling in B6-BMDM infected with *L. amazonensis* [28]. However, the association between miRNAs and *Leishmania* diseases will only be fully understood once the genetic background is complete as well as when we have an undertsanding of the parasite species that could interfere in the post-transcriptional regulation mediated by miRNAs.

Once the modulation of *Arg*1 and *Nos*2 as well as *Tnfa* was implicated in the activation of the macrophage inflammatory response and the control of the parasite infection [7], we predicted that the miRNA interactions would be focused on the genes involved or correlated to arginine metabolism, which is implicated in amino acid transport (*Cat*1/*Slc*7*a*1 and *Cat*2/*Slc*7*a*2*)*, polyamine production (*Arg*12 *Arg*22 and *Odc*1), and NO production (*Nos*2) (Figure 1). We found that miR-294-3p interacts with *Nos*2 (as previously validated) and predicted the targets of *Cat*1/*Slc*7*a*1 and *Cat*2/*Slc*7*a*2; miR-301b-3p can predict the targets of *Cat*1/*Slc*7*a*1 and *Arg*2; and miR-410-3p can predict the targets *Cat*1/*Slc*7*a*1 and *Nos*2. The analysis of the quantities of target genes and miRNAs in B6-macrophages infected with *L. amazonensis* showed lower *Cat*1/*Slc*7*a*12 *Cat*2/*Slc*7*a*22 *Nos*22 *Arg*12 and *Odc*1 and increased levels of miR-294-3p, miR-301b-3p, and miR-410-3p. miR-301b-3p and miR-410-3p are not modulated in BALB/c-macrophages infected with *L. amazonensis*, but *Cat*1/*Slc*7*a*12 *Cat*2/*Slc*7*a*22 and *Arg*1 are expressed [27]. Moreover, miR-294 comprises a miR-290-295 cluster and regulates the cell cycle during embryogenesis [43,44]. The miR-379/miR-410 gene cluster is a large genomic miRNA cluster that is involved in various aspects of neurodevelopment and neuronal maturation [45].

The availability of arginine, a common substrate to ARG1 and NOS2, can regulate the levels of CAT1 and CAT2 transporters in the external plasmatic membrane and the parasitophorous vacuole [19,46,47]. This complex regulation in arginine uptake is one of the multiple factors involved in regulating production during *Leishmania* infection through *Nos*2 transcription and NOS2 translation and through the presence of the substrates and co-factors such as O2, biopterin, NADPH, and others [48]. As previously shown, the B6-macrophages only increased *Nos*2, NOS2, and NO production when miRNA let-7e was inhibited during *L. amazonensis* infection, reducing the infectivity [28]. The IL-1β and TNF levels also induced NO production [49]. Our group showed increased levels of arginine, citrulline, and polyamines inside the macrophages infected with *L. amazonensis* [50]. These data increased the modulation expectation of genes that can compete for arginine to produce polyamines, such as C*at*s, *Arg*1, *Arg*2, and *Odc*1. Although the levels of *Cat*1/*Slc*7*a*12 *Cat*2/*Slc*7*a*22 *Arg*1, and *Odc*1 did not behave as expected during infection, increased levels of the *Arg*2 transcript level were not reflected by the increased competition of cytoplasmatic arginine with NOS2 because of its mitochondrial localization. Further, the regulation of arginase 2 by miR-155 regulates the arginine availability in the DCs, allowing t-cell activation [51]. Il-10 and *Arg*2 synergy works to reprogram the immunometabolic response of macrophages in response to LPS, reducing the levels of succinate, HIF-1α, and IL-1β [52].

Interestingly, in LPS stimulation, we found an inversion of the phenomenon observed in infected macrophages, proving an increased level of the target genes *Cat*1/*Slc*7*a*12 *Cat*2/*Slc*7*a*22 *Nos*22 *Arg*12 *Arg*22 and *Odc*1 and reduced levels of the miRNAs miR-294-3p, miR-301b-3p, and miR-410-3p, excluding these miRNAs from the inflammatory macrophage phenotype. LPS induces *Cat*2, *Arg*1, and *Nos*2-NO in macrophages, suppressing T cell proliferation through the starvation of arginine and causing glycolysis dependence [53]. This fact guided us to only study the functional inhibition of miRNA in infected macrophages so that we could understand the miRNA impacts in the B6-macrophages.

The reduction in the levels of miR-294, the maintenance of miR-301b, and the maintenance/reduction of miR-410 in the functional inhibition assay showed difficulty and variability in the efficiency in the quantification of miRNA during the inhibition assay, as previously observed in our own studies as well as in those of others [27,28,38,54]. Nevertheless, the modulation of mRNA levels can help us study miRNA interactions during infection by considering the post-transcriptional and translational mechanisms of gene expression regulation that interfere with the mRNA and protein levels as well as the stability and cellular compartmentalization of proteins [5]. The functional inhibition of miR-294 and miR-410 increased the *Cat*2/*Slc*7*a*2 and *Cat*1/*Slc*7*a*1 transcripts, respectively, which implies the increase of protein translation and arginine uptake. Kishikawa et al. showed that miR-122 represses CAT1 and regulates arginine availability [55]. miR-294 and miR-301b inhibition at 4 h of infection tended to increase *Nos*2 levels and reduced them at 24 h. The amastigote number and infectivity index reduced the inhibition of miR-294. Similarly, *Nos*2 transcripts were maintained during the inhibition of miR-294 in infected BALB/c-macrophages. Only the NOS2 protein levels increased in this condition [27].

The differential expression of miRNA, previously observed in B6-macrophages in the network regulation of signaling pathways mediated by TLR recognition, supports these ideas [28]. In a RAW 264.7 macrophage lineage model, the mimetics of miR-294 using mimics during LPS stimulation reduced the protein levels of TNF-α and IL-6 due ti miR-294 targeting TREM-1 [56]. We also showed that miR-294 inhibition increased *Tnfa* levels after 24 h in infected B6-macrophages. Indeed, miR-301b is associated with NF-κB activity inhibiting p65 nuclear translocation in pancreatic Panc-1 and BxPC-3 cell lines, interfering with LPS signals, and corroborating the involvement of these miRNAs in the regulation of inflammation [57], which is implicated in TNF and NOS2 expression. Intriguingly, miR-130b/miR-301b belongs to the same cluster family, sharing target genes and cell proliferation with prostate cancer [58]. miR-301b inhibition did not interfere in the *Tnfa* transcripts even though miR-301b shares the same seed as *Tnfa* 3′UTR does with miR-294, which is probably because it acts in a different way in the absence of miR-294.

Zuo et al. elegantly demonstrated the lower levels of miR-410-3p and increased the predicted target TLR2 in LPS-induced sepsis in mice, where miR-410-3p overexpression mediated TLR2 inhibition, relieving mitochondrial dysfunction and chemokine production [59]. miR-410 mimics also reduced IL6 and TNF levels in LPS-stimulated chondrocytes [60].

Overall, the present study showed that *Leishmania* subverts the macrophage–miRNA profile, altering miR-294, miR-301b, and miR-410 expression and the transcripts of the target genes *Cat*2*/Slc*7*a*2, *Cat*1/*Slc*7*a*12 *Nos*2, and *Tnfa*, building a complex network of miRNAs–mRNAs modulated during the recognition and activation of macrophages, leading to an infection outcome. Based on our findings, we speculate whether we may consider miR-294 and miR410 as a biomarker of *L. amazonensis* infection and for the development of therapeutic formulations for cutaneous leishmaniasis.

## 4. Materials and Methods

### 4.1. In Silico miRNA Binding Site Prediction

The interaction between miRNAs and mRNAs was assessed the using the miRMap search tool [35]. All searches were made by setting *Mus musculus* as the species parameter using a combination of open ΔG > 9 (mRNA opening free energy), exact probability > 60 (exact distribution of site over-representation probability), and miRmap score > 60 using Vienna-RNA algorithm [61].

### 4.2. Parasite Culture

*Leishmania amazonensis* (MHOM/BR/1973/M2269) promastigotes were cultivated at 25 °C in M199 culture media (Invitrogen, Grand Island, NY, USA) supplemented with 10% heat-inactivated FBS (Invitrogen), 5 ppm hemin, 100 μM adenine, 50 U penicillin, 50 μg/mL streptomycin (Invitrogen), 40 nM Hepes-NaOH buffer, and 12 mM NaHCO_3_ at a pH of 6.85. The culture was split every 7 days with preset inoculum 1 × 10^6^ cells per 10 mL and was maintained for up to five passages. Neubauer’s chamber determined the number of cells, diluting 20× the cell suspension in phosphate-buffered saline (PBS 1×) plus 1% formaldehyde.

### 4.3. BMDM Harvesting and Culture

C57BL/6. Tac animals were purchased from the Medicine School of the University of São Paulo at 6 to 8 weeks old. They were euthanized inside a CO_2_ chamber. Afterward, bone marrow was extracted from the femurs, tibias, and humeri by flushing them with ice-cold PBS 1× with a 24 G × 3/4 needle. The cell suspension was separated with a 21 G × ½ needle followed by centrifugation at 4 °C at 500× *g* for 10 min. The cells were resuspended in complete RPMI 1640 (LGC Biotechnologies, São Paulo, Brazil) supplemented with 10% heat-inactivated FBS (Gibco, Thermo Scientific, Waltham, MA, USA), 0.5% Pen/Strep (1 U/mL), 2-mercaptoethanol (50 μM), L-glutamine (2 mM), sodium pyruvate (1 mM), and 10% L9-29 supernatant as a source of Macrophage Colony-Stimulating Factor (M-CSF). The cells were cultivated for 7 days at 34 °C in 5% CO_2_.

### 4.4. Macrophage Infection

After the macrophage differentiation, cells were seeded into 24-well plates (1 × 10^6^ cells/well; SPL, Lifescience, Pocheon, Korea) or 8-well chamber slides (5 × 10^4^ cells/well; Millipore, Merck, Darmstadt, Germany). Then, stationary phase promastigotes were added into the wells at MOI 5:1. After 4 h, the cells were washed two times with PBS 1× at room temperature to clean the unphagocytosed parasites. The culture was maintained for 4 and 24 h. The cells were stimulated with LPS (100 ng/mL; *Escherichia coli* LPS serotype 0127:B8, Sigma-Aldrich, St. Louis, MO, USA) to induce M1-inflammatory macrophages.

### 4.5. Infection Assessment

After 4 and 24 h of infection, the chamber slides were washed two times with PBS 1× and were then fixed with acetone/methanol (1:1 *v*/*v*; Sigma-Aldrich, St. Louis, MO, USA) and were incubated at −20 °C for 24 h followed by Giemsa staining (Fast Panotic Kit, Laborclin, São Paulo, Brazil). At least 600 macrophages were counted per well to achieve the parameters of the Macrophage Infection Rate [(infected macrophage/total macrophage) ∗ 100], amastigotes per infected macrophage, and the infection index, which was calculated by multiplying the rate of infected macrophages by the average number of amastigotes per macrophage.

### 4.6. RNA Extraction and cDNA Preparation

For RNA extraction, macrophages (3 × 10^6^) were mixed with 750 μL of Trizol (Ambion, Thermo Scientific, USA) and 250 μL PBS 1×. Then, chloroform was added to separate the aqueous and organic phases. Following the manufacturer’s instructions, the aqueous phase was added into miRNeasy (Qiagen, Hilden, Germany) columns. The extracted RNA was measured using Nanodrop (ND-100, Thermo Scientific) to reach a 200 ng/μL RNA solution. The total RNA was used to produce complementary DNA (cDNA) of mature mRNA and miRNA. For miRNA, we used the miScript II RT kit (Qiagen, Hilden, Germany), following the manufacturer’s recommendations and starting from 250 ng of RNA. To mature the mRNA, starting from 2 μg of RNA, we used random hexamer primer (1.5 μg/mL; Thermo Scientific), dNTP (10 μM; Fermentas, Thermo Scientific), RevertAid Reverse Transcriptase (200U; Thermo Scientific), and RiboLock (Thermo Scientific) to a final volume of 40 μL. The cycling protocol was used following the manufacturer’s recommendations. All cycles were undertaken using the Mastercycler Nexus Gradient (Eppendorf, Hamburg, Germany).

### 4.7. Transcript Quantification

A quantitative PCR was used to assess the amount of mRNA, which was conducted using LuminoCt SYBR Green qPCR Ready Mix (Sigma-Aldrich), specific primers (0.1 μM), and a cDNA sample (5 μL, 100-times diluted) to a final volume of 10 μL. The reaction was performed in the QuantStudio Thermocycler (Thermo Scientific) following the following protocol: 94 °C to the initial denaturation, 40 cycles of denaturation (94 °C, 30 s), annealing and extension (60 °C, 30 s), and fluorescence capture at the end of every cycle. After 40 cycles, a melting curve was found between 60 °C and 95 °C, with fluorescence registers at every 0.5 °C. The primer list was as follows: *Nos*2-F: 5′-agagccacagtcctctttgc-3′ and *Nos*2-R: 5′-gctcctcttccaaggtgctt-3′; *Arg*1-F: 5-agcactgaggaaagctggtc-3′ and *Arg*1-R: 5′-cagaccgtgggttcttcaca-3′; *Arg*2-F: 5′-tctcctccacgggcaaattc-3′; *Arg*2-R: 5′-cactcctagcttcttctgccc-3′; *Cat*-2/*Slc*7*a*2: 5′-tatgttgtctcggcaggctc-3′ and 5′-gaaaagcaacccatcctccg-3′; *Cat*1/*Slc*7*a*1: 5′-cgtaatcgccactgtgacct-3′ and 5′-ggctggtaccgtaagaccaa-3′; *Tnfa*-F: 5′-ccaccacgctcttctgtcta-3’; *Tnfa*-R: 5′-agggtctgggccatagaact-3’;and *β-*2*-microglobulin*-F: 5′-cactgaattcacccccactga-3′, and *β-*2*-microglobulin*-R: 5′-acagatggagcgtccagaaag-3′; *Odc*1-F: 5′-ctgccagtaacggagtccag-3′*Odc*1-R: 5′-tcagtggcaatccgtagaacc-3′. The fold-change was calculated using the Delta–Delta Ct (∆∆Ct) method and was based on β-2-microglobulin and was normalized with the uninfected group or negative control. The fold-change was presented in log2 (Table 1).

To assess the amount of miRNA, the specific amplification of miR-294-3p, miR-301b-3p, miR-410-3p, and SNORD95A (used in normalization) was completed by qPCR, which was performed with an miScript SYBR Green (Qiagen), 10× miScript Universal Primer, 10× Specific Primer, and 2 μL of cDNA (diluted 10-fold). RNAse-free water was added to a final volume of 10 μL. The reaction was performed in the QuantStudio Thermocycler (Thermo Scientific) with the following protocol: activation of the HotStart DNA Polymerase for 15 s at 95 °C and 40 cycles of 15 s at 94 °C followed by 30 s at 55 °C and 30 s at 70 °C. The fold-change was calculated using the Delta–Delta Ct (∆∆Ct) method and was based on SNORD95 and was normalized with the uninfected group or negative control. The fold-change was presented in log2.

### 4.8. miRNA Inhibition

The miR-inhibition was performed with the addition of 50 nM of miR-294-3p-, miR-301b-3p-, or miR-410-3p-specific inhibitors (cat 4464084) or the negative control (cat 4464076) (miRVana, Ambion, Thermo Scientific) mixed with 6 μL FuGene Reagent Transfection (Roche, Promega, Madson, WI, USA), which was incubated for 20 min at room temperature following our previously described protocol [62] in 500 μL of serum-free RPMI 1640 medium (LGC Biotecnologia, São Paulo, SP, Brazil) [27]. After 24 h of transfection, the cells were infected as described above.

### 4.9. Ethics Statement

The experimental protocol for the animal experiments was approved by the Comissão de Ética no Uso de Animais (CEUA) from the Instituto de Biociências of the Universidade de São Paulo (approval number CEUA-IB: IB-USP 233/2015). This study was conducted in strict accordance with the recommendations in the guide and policies for the care and use of laboratory animals of São Paulo State (Lei Estadual 11.977, de 25 August 2005) and the Brazilian government (Lei Federal 11.794, de 8 October 2008).

## Figures and Tables

**Table 1:** Primer sequences for qPCR.

Target Gene		Primer (5′-3′)
Nitric Oxide Synthase (*Nos*2)	Forward	5′-agagccacagtcctctttgc-3′
	Reverse	5′-gctcctcttccaaggtgctt-3′
Arginase 1 (*Arg*1)	Forward	5′-agcactgaggaaagctggtc-3′
	Reverse	5′-cagaccgtgggttcttcaca-3′
Arginase 2 (*Arg*2)	Forward	5′-tctcctccacgggcaaattc-3′
	Reverse	5′-cactcctagcttcttctgccc-3′
Cationic amino acid transporter 1 (*Cat*1/*Slc*7A1)	Forward	5′-cgtaatcgccactgtgacct-3′
	Reverse	5′-ggctggtaccgtaagaccaa-3′
Cationic amino acid transporter 2 (*Cat*2/*Slc*7A2)	Forward	5′-tccaaaacgaagacaccagt-3′
	Reverse	5′-gccatgagggtgccaataga-3′
Tumor Necrosis Factor alpha (*Tnfa*)	Forward	5′-ccaccacgctcttctgtcta-3′
	Reverse	5′-agggtctgggccatagaact-3′
Ornithine decarboxilase (*Odc*1)	Forward	5′-ctgccagtaacggagtccag-3′
	Reverse	5′-tcagtggcaatccgtagaacc-3′
β-2-microglobulin (*B*2*m*)	Forward	5′-cactgaattcacccccactga-3′
	Reverse	5′-acagatggagcgtccagaaag-3′
Glyceraldehyde-3-Phosphate Dehydrogenase (*Gapdh*)	Forward	5′-ggcaaattcaacggcacagt-3′
	Reverse	5′-ccttttggctccacccttca-3′

## Data Availability

Not applicable.
